# Adolescents’ Life Satisfaction, Physical Activity, and the Moderating Role of Gender: A Cross-Country, Multilevel Analysis in 64 Countries

**DOI:** 10.3390/children12101375

**Published:** 2025-10-11

**Authors:** Carmel Cefai, Beatriz Barrado, Gregorio Gimenez, Valeria Cavioni

**Affiliations:** 1 Department of Psychology, Faculty for Social Wellbeing, University of Malta, MSD 2080 Msida, Malta; 2 Departament of Economics and Statistics, Faculty of Economics and Business Administration, University of León, Campus de Vegazana, 24071 León, Spain; beatriz.barrado@unileon.es; 3 Faculty of Economics and Business, University of Zaragoza, Gran Vía 2, 50005 Zaragoza, Spain; 4 Department of Humanities and Social Sciences, Faculty of Society and Communication Sciences, Universitas Mercatorum, 00186 Rome, Italy; valeria.cavioni@unimercatorum.it

**Keywords:** subjective well-being, life satisfaction, physical activity, adolescence, PISA, cross-country studies

## Abstract

**Highlights:**

**What are the main findings?**

• This study finds a positive and significant association between physical activity and life satisfaction across 64 countries
• For most countries, we did not find a significant gender moderator effect.

**What are the implications of the main findings?**

• We provide strong evidence for the potential of physical activity as a universally beneficial intervention to improve adolescents’ well-being.
• Physical activity may be considered as a Positive Childhood Experience (PCE) in the healthy development of adolescents

**Abstract:**

**Background:** Engaging in physical activity (PA) is especially significant for adolescents, as this is a key developmental stage for establishing lifelong habits. While the physical, mental, and cognitive health benefits of PA are well-documented, less is known about its relationship with adolescents’ life satisfaction (LS). Most existing evidence often involves small sample sizes, focusing particularly on developed regions, and few studies use large-scale comparative data. **Methods:** This study examines the association between adolescents’ LS and PA using data from the 2022 Programme for International Student Assessment (PISA), the world’s largest comparative education survey of adolescents. Our analysis included 399,794 adolescents from 64 high- and middle-income countries and economies. We used three-level multilevel regressions. **Results:** We found that, after controlling for individual, family, and school factors, PA is positively and significantly associated with LS. This finding holds for the pooled sample and across the 64 countries analysed. For most countries, we did not find a significant gender moderator effect, suggesting that the positive association between PA and LS did not vary by gender. **Conclusions:** The findings suggest a global health promotion strategy to promote PA amongst adolescents as a normative developmental process necessary for their well-being and mental health.

## 1. Introduction

Adolescence represents a critical period characterized by rapid developmental transitions, growth, and the emergence of self-identity, ultimately leading to the onset of emerging adulthood. This pivotal developmental stage is frequently accompanied by a range of challenges, including academic stress, exposure to bullying and cyberbullying, problematic internet use, loneliness, mental health concerns, environmental anxieties, socio-economic disadvantages, and social exclusion. These stressors contribute to a complex landscape that can significantly impact adolescents’ psychological well-being, at a time when around half of mental health issues develop before the age of 18 [1].

UNICEF [2] conceptualises children and adolescents’s well-being both in terms of health, safety, material security and education as well as their socialization, and their sense of being loved, valued, and included in the families and societies, putting particular emphasis on context and children’s experiences. Current research on well-being in children and adolescents posits that well-being is a multi-dimensional construct, encompassing both objective indicators—such as access to education, health care, welfare, and protection, and subjective components, including perceived quality of life and life satisfaction [3,4]. Recently there has been increasing interest in the subjective well-being and life satisfaction of children and adolescents. First, objective well-being on its own, such as measures of health, education and economic wellbeing, provides an inadequate understanding of children’s and adolescents’ wellbeing needs [5]. In order for policies to be effective, they need to take into consideration their own understandings of well-being and life satisfaction [6]. How children and adolescents construe their well-being may vary from adults’ conceptualisation, underlining such factors as satisfaction, happiness and relationships, in contrast to more objective indicators mentioned by adults (e.g., [4,5]). Furthermore, research from developmental sciences, education and other disciplines underline children and adolescents as social actors engaged in shaping their own lives, and thus researchers need to engage with them in advancing the field [7]. This reflects the children’s rights approach, instigated by the UN Convention for the Rights of the Child, with Article 12 in particular advocating for children’s right to participate and express their views on policies, practice and research about themselves.

Life satisfaction (LS) is a commonly used indicator of subjective well-being among young people. It involves adolescents’ cognitive evaluations of their quality of life, both globally (overall LS) and across specific domains, such as family, school, community, friendship, use of time, health, future outlook, and self-concept [4]. In this paper we focus on LS as a key aspect of subjective well-being, whilst acknowledging that well-being includes other domains as well, such as psychological and affective well-being.

Over the past two decades, there has been a documented decline in life satisfaction and an increase in loneliness among adolescents, with a notable disparity between childhood and adolescence [3,8]. It is indicative that globally subjective well-being and LS decline from late childhood to adolescence and young adulthood [9,10]. There are also indications in various countries across the world that adolescent subjective well-being has been declining both before and after the COVID-19 pandemic, with such declines being more marked amongst females, the only exception being East Asia, with the PISA results indicating a post-pandemic increase in the LS of adolescents [11]. There is also a marked gender difference in adolescent LS, with 15-year-old females reporting lower LS [12,13,14,15]. This gender difference has become more marked in recent years as indicated by both PISA [3] and WHO HBSC studies [8].

Socio-economic status is another key determinant of life satisfaction, with adolescents from more affluent families reporting higher levels of LS and well-being, highlighting the influence of socio-economic factors, such as material conditions, on adolescent development and quality of life [16,17]. Country economic development has been identified as a crucial determinant of adolescent well-being and LS [11]. Similarly, coming from a migrant background has been associated with lower LS [16,17,18]. However, LS in adolescence is determined by both socio-economic conditions as well as social and cultural contexts, such as family and peer relationships and school processes [4,11,19].

In educational settings, teachers and peers play a critical role in student well-being and LS [4,20]. Empirical evidence has shown that high levels of school and peer connectedness act as protective forces for young people, contributing positively to LS [21]. Schools with supportive teachers who build positive relationships with their students, care about them and foster an engaging atmosphere are essential for promoting positive relationships and fostering adolescents’ LS [20,22]. Conversely, poor relationships and bullying victimisation are negatively related to LS [23,24,25].

### Physical Activity and Its Impact on Adolescent Well-Being and Life Satisfaction

In recent years, a substantial body of research has emerged, focusing on the promotion and enhancement of adolescent well-being, with particular attention to identifying the processes that contribute to LS [3,4]. Among these factors, the role of physical activity (PA) in influencing LS, well-being, and mental health has garnered increasing interest in adolescent psychology. WHO [26] defines PA as “any bodily movement that increases energy expenditure above resting energy expenditure”. This definition encompasses a wide range of activities, including physical exercise and sports, which are typically planned, structured, and may also involve competitive elements.

Extensive research has explored the relationship between physical activity and both physical and mental health in children and adolescents.

Numerous studies have demonstrated that PA confers a variety of mental health benefits, such as reduced anxiety and depression, enhanced self-esteem, well-being, LS, and increased positive behaviours. Eime et al. [27] conducted a systematic review of 30 studies examining the psychological and social benefits of sports participation among children and adolescents. Their findings indicated that participation in sports is associated with improved self-esteem, enhanced social interactions, and reduced depressive symptoms. The review also found that team sports were more strongly associated with positive health outcomes than individual sports. Similar findings were reported by Spruit et al. [28] in a meta-analytic review of 57 studies investigating the overall effects of PA interventions amongst adolescents. The review revealed that PA contributes to reductions in internalising and externalising problems while enhancing self-concept and academic performance.

More recent research by Rodriguez-Ayllon et al. [29] confirmed the relationship between PA and well-being and mental health in children and adolescents. Their meta-analysis that incorporated over 100 cross-sectional and longitudinal studies demonstrated a significant relationship between PA and reduced psychological ill-being (e.g., depression, negative affect), as well as increased psychological well-being (e.g., self-concept, LS). Conversely, sedentary behaviours such as prolonged television watching were associated with heightened psychological ill-being and diminished LS. The study further revealed that adolescents who engaged in at least one hour of physical exercise per day experienced more pronounced mental health benefits than those who engaged in less than an hour of exercise daily.

The existing literature consistently highlights the positive impact of PA—whether individual or social, structured or unstructured, and encompassing active mobility—on the well-being and LS of children and adolescents. Whilst PA has a positive impact on well-being and LS, it could also be argued, however, that a sense of wellbeing and life satisfaction may make it easier for adolescents to engage in PA. However, longitudinal studies clearly indicate that PA contributes to subjective wellbeing, life satisfaction and mental health [27,28,29,30]. In this study we argue that PA promotes LS and well-being through various biopsychosocial processes, such as neurobiological changes in the brain, behaviour change such as enhanced sleep and self-regulation, enhanced cognitive functions, improved self-concept and self-esteem, and satisfaction of the psychological needs of relatedness, competence and autonomy [31,32]. These biopsychosocial pathways then lead to improved life satisfaction and sense of well-being, enhanced academic learning and performance, and reduced mental health issues such as anxiety and depression [27,28,29,33,34,35,36,37,38].

## 2. Present Study

Despite these positive findings on the relationship between PA and LS and well-being, there remains a significant gap in high-quality research, with many studies exhibiting small overall effect sizes [29,37]. Furthermore, most research has focused on mental health conditions rather than broader constructs such as well-being and LS (e.g., [28,29,37]). There is also a lack of global data on well-being in early-to-middle adolescence (age 10–15), with many world regions having no available information. While there is considerable research on adolescent subjective well-being within specific cultural contexts, international and global research remains limited [11]. Furthermore, adolescents have also been identified as an understudied population in PA research [38] and in studies investigating PA and LS [39,40].

This study addresses the following two research questions: RQ1. Is PA positively and significantly associated with LS among adolescents? RQ2. Does this relationship between PA and LS significantly differ between males and females?

It examines these questions using global data from the PISA 2022 dataset. While there is some evidence on the positive link between PA and LS among adolescents, previous studies have produced limited evidence for several reasons. First, studies involving adolescents often rely on small sample sizes, focus on developed regions, and few use large-scale comparative data. The limited cross-national studies available have typically been conducted in a reduced number of countries, predominantly in North America and Western Europe [11,41]. Second, most of the studies used datasets that did not allow for control of important confounders, such as students’ feelings and school characteristics (e.g., bullying victimisation, climate), which are determinants of adolescent LS [22,24]. Third, most of the studies have not explored gender disparities in the link of PA and LS, although several studies have found that females engage in less PA [42,43] and report lower levels of LS [13,15]. Identifying gender differences in the effect of health-enhancing behaviors such as PA on well-being outcomes remains an important goal of public health [42] and is key to designing effective interventions. The literature has explicitly called for international studies that cover a wider range of countries and examine these differences by gender [41]. Finally, there is a lack of cross-country studies analysing the relationship between PA and LS and the moderating role of gender on a country-by-country basis. Providing evidence for these relationships by country is essential to identifying national differences and designing effective intervention Programmes accordingly.

To address these research gaps, our study aims to examine the link between PA, LS, and the moderating role of gender using the PISA 2022 survey, the world’s largest comparative education survey for adolescents. PISA contains comparable information on 15-year-old students from countries in Europe, the Americas, Oceania, Asia, and the Middle East. The detailed information about students’ contexts enabled us to control for several socio-demographic and school characteristics related to LS. Importantly, PISA’s homogeneous approach, which ensures comparability across countries, allows us to examine the relationship between LS, PA, and the moderating role of gender both in the pooled sample and by country. To the best of our knowledge, this study is the first empirical analysis of these relationships using the full PISA survey, and it allows us to benefit from a sample that includes a large number of countries and adolescents. The combination of broad geographic scope and detailed student data provides a valuable contribution to advancing our understanding of the links between PA, LS, and gender differences across diverse contexts.

## 3. Methodology

### 3.1. Data and Participants

The data came from the 2022 PISA wave. PISA is a worldwide triennial project by the Organisation for Economic Co-operation and Development (OECD). This cross-sectional survey focused on 15-year-old students’ academic performance. It also collects information on students’ backgrounds, home life and habits, attitudes toward school and teachers, and learning experiences [44]. Since the 2015 wave, PISA has also offered data on students’ well-being and, for the first time, in 2022, an index of PA. In the 2022 wave, 613,744 students completed the assessment in 81 participating countries/economies.

The PISA sample selection follows a two-stage stratified design. In the first stage, at least 150 schools in each participating country or economy, were selected. When the total number of schools was below this threshold, all schools were included. Within every selected school, a fixed number of students (42 for the computer-based assessment and 35 in the case of paper-based format) was randomly chosen with equal probability. In systems with smaller total student populations, all students were selected. Importantly, PISA establishes that the target cluster size could not fall below 25 students, in order to maintain sufficient precision in estimating of variance components across both the within- and between-school dimensions [44].

Our sample was restricted to participants with complete responses for all variables included in our model. For the present analysis, we used data from 399,794 students (253,169 girls and 246,263 boys) who studied in 16,997 schools across 64 countries and territories worldwide. Table A1 in Appendix A lists the participating countries and territories.

### 3.2. Measures

In this section, we present the variables included in the model.

Life satisfaction (dependent variable) was measured by the question “How satisfied do you feel about your life, on a scale from 0 (“not at all satisfied”) to 10 (“completely satisfied”). We focus exclusively on Life Satisfaction given that, unfortunately, PISA 2022 does not provide other measures of subjective well-being. Although single items are often criticised, the use of the single-item Life Satisfaction measure is well established in the literature, being a common approach in empirical research on adolescents’ subjective well-being (see, for instance, [14,24,45,46]) and its validity has been supported by a number of studies [47,48]. Moreover, the OECD has also emphasised its simplicity and convenience for cross-cultural samples [49,50,51] as it offers an acceptable balance between practical needs and psychometric considerations.

Physical activity (key independent variable) was measured by the PISA index, which was derived from student responses on how many days during a typical school week they exercised or practised a sport (e.g., running, cycling, aerobics, soccer, skating, country-specific sport) before going to school and/or after leaving school. Responses were scaled into the index of “Exercise or practise a sport before or after school”. Each item included six response options (“0 days”, “1 day”, “2 days”, “3 days”, “4 days”, “5 or more days”). Values on this index range from 0 (no exercise or sports) to 10 (engaging in exercise or sport 10 or more times per week).

Additionally, an interaction term (*Male × PA*) was created with our key independent variable (PA) and the dummy variable male (equal to 1 whether the student was a male student, and 0 otherwise) to explore if the relationship between LS and PA differed for male and female students (moderator variable).

The covariates (control variables) were selected from the literature on the determinants of adolescent LS. All variables, except the GDP per capita provided by the World Bank, came from the PISA 2022 dataset. Table A2 in Appendix A provides a detailed definition of the variables. All index variables were constructed and provided by PISA. Cronbach’s alpha was calculated by PISA to assess the internal consistency of each index within countries, ensuring cross-country comparability. In all cases, Cronbach’s alpha values ranged between 0.7 and 0.9, indicating satisfactory to high levels of internal consistency [44]. Table A3 in Appendix A reports the country-level Cronbach’s alpha values for the constructed indices derived from IRT scales.

The individual and family characteristics included age, gender, country of birth, economic, social and cultural status. Our model also controlled for social relationships and the school environment, namely being bullied, disciplinary climate, teacher support, and quality of student–teacher relationships. At the country level, we controlled for the GDP per capita in 2022 and adjusted for purchasing power parity in constant 2017 dollars [52].

### 3.3. Analysis

We estimated Hierarchical Linear Models (HLM) with three levels, namely student, school, and country. Students’ LS was regressed on the key variable, PA and a set of students’ and school characteristics that we used as control variables. HLM, commonly adopted in PISA studies, considers the hierarchical design of PISA, where students (level 1) are nested within schools (level 2), and schools are nested within countries (level 3). Due to this nested structure, observations tend to be dependent because of the shared environment [53]. HLM offers statistical advantages over traditional regression methods (e.g., OLS regression) by explicitly modelling the dependency correlations within the same school and country, resulting in unbiased standard errors and efficient estimates [54]. To carry out the estimations, it is necessary to homogenise the information at the same level; in our case, we used the student level, which allowed us to maximise the number of observations and to have more estimation power.

Our analytical approach was structured in two phases. First, we used the pooled sample to estimate the relationship between LS and PA, as well as the moderating role of gender. Second, we replicated the estimations separately by country. The regressions were conducted using Stata 18 statistical software [55].

## 4. Results

### 4.1. Descriptive Statistics

Table 1 displays the descriptive statistics for the pooled sample of countries. The average *LS* was 6.902 (6.569 for females and 7.243 for males), ranging from 0 to 10, with a standard deviation (SD) of 2.654. The average times of *PA* per week was 0.527 (3.676 for females and 5.409 for males), ranging from 0 to 10, with an SD of 3.626. Table 2 presents the average values for *LS* and *PA* disaggregated by gender and country. In most countries (53 out of 64), males reported higher *LS* scores than females. Additionally, we observed notable differences in *PA* by gender, with males in nearly all countries (61 out of 64) reporting more times of *PA* per week than females.

Table A4 in Appendix A presents the correlations between the variables. The correlation between *LS* and *PA* was positive and significant (*p* < 0.01), with a value of 0.16. The correlation coefficients among the predictors were significant and ranged from −0.22 to 0.35 (exhibiting the expected signs). As all the values were below the 0.80 threshold, there were no signs of collinearity [56]. Variance Inflation Values (Table A5 in Appendix A) also corroborated the lack of collinearity issues.

### 4.2. Estimations for the Pooled Sample of Countries

#### 4.2.1. Is *PA* Positively and Significantly Associated with *LS* Among Adolescents? (RQ1)

Table 3 presents the results for the pooled sample. Column 1 shows the results for the null model. The intra-class correlation (ICC) values provide an estimate of the proportion of variance in students’ *LS* explained at the school and country levels. ICC values showed that 4.5% of the variance in students’ *LS* can be attributed to differences between schools and 14.6% to differences between countries. This substantial variation at both the school and country levels underscores the necessity of applying three levels HLM.

Column 2 shows the estimations of the baseline regression. Results showed that *PA* was positively and significantly related to *LS* after controlling for our wide set of student and school characteristics affecting *LS* (*p* < 0.01).

The results also show that being male was positively and significantly correlated with *LS* (*p* < 0.01). *Age* was negatively and significantly correlated with *LS* (*p* < 0.01). *Foreigners* reported lower levels of *LS* (*p* < 0.05). *Economic, social, and cultural status* was positively and significantly correlated with *LS* (*p* < 0.01). *Being bullied* was negatively and significantly correlated with *LS* (*p* < 0.01). *Disciplinary climate*, *teacher support*, and *quality of student–teacher* relationship were positively and significantly correlated with *LS* (*p* < 0.01). Finally, *GDP per capita* was negatively and significantly correlated with *LS* (*p* < 0.01).

#### 4.2.2. Does This Relationship Between *PA* and *LS* Significantly Differ Between Males and Females? (RQ2)

Column 3, in Table 3, incorporates an interaction term (*Male × PA*) to explore whether the relationship between *LS* and *PA* differs between male and female students. The coefficient of the interaction term was not significant, indicating that the positive association between *PA* and *LS* did not vary by gender.

### 4.3. Estimations by Country

#### 4.3.1. Is *PA* Positively and Significantly Associated with LS Among Adolescents? (RQ1)

Table 4 and Table 5 report the estimations for each of the 64 countries separately. Table 4 shows the estimations of the *PA* coefficients once we controlled for all the model predictors. The findings were consistent: *PA* was positively and significantly associated with *LS* (*p* < 0.01) across all 64 countries analysed.

#### 4.3.2. Does This Relationship Between PA and LS Significantly Differ Between Males and Females? (RQ2)

Table 5 reports the coefficients for the moderating effect of gender once we controlled for all the model predictors. Our findings indicated that, with few exceptions, the coefficient of the interaction term (*Male × PA*) was not significant, suggesting that the positive association between *PA* and *LS* did not vary by gender. Specifically, in 55 out of 64 countries, there were no significant differences in the relationship between *LS* and *PA*. On the other hand, in Brazil and Germany, the positive relation between *PA* and *LS* was more pronounced for male than female students, while conversely, in Finland, Angola, Saudi Arabia, Spain, and the United Arab Emirates, the positive relationship between *PA* and *LS* was stronger for female than male students.

**Table 5:** The Moderating Role of Gender. Analysis by Country.

Country	Coeff.	Std. Err.	Country	Coeff.	Std. Err.
Albania	−0.035	(0.023)	South Korea	0.009	(0.021)
Qatar	0.018	(0.028)	Latvia	−0.041 **	(0.020)
Argentina	0.003	(0.024)	Lithuania	−0.005	(0.019)
Austria	−0.009	(0.023)	Malta	0.025	(0.025)
Brazil	0.029 *	(0.017)	Mexico	−0.002	(0.021)
Bulgaria	0.007	(0.022)	Mongolia	0.001	(0.017)
Chile	−0.034	(0.025)	Moldova	0.018	(0.023)
Chinese Taipei	0.007	(0.018)	Montenegro	−0.010	(0.025)
Colombia	0.000	(0.020)	Netherlands	−0.002	(0.021)
Croatia	0.021	(0.017)	New Zealand	−0.002	(0.022)
Czech Republic	0.006	(0.020)	Panama	0.031	(0.036)
Denmark	−0.030	(0.022)	Paraguay	0.005	(0.024)
Dominican Republic	−0.005	(0.021)	Peru	0.002	(0.021)
El Salvador	0.038	(0.025)	Poland	−0.006	(0.026)
Estonia	−0.035 *	(0.021)	Portugal	0.021	(0.017)
Finland	−0.059 ***	(0.015)	Qatar	0.021	(0.027)
France	0.012	(0.020)	Romania	0.013	(0.017)
Georgia	0.010	(0.020)	Saudi Arabia	−0.037 *	(0.021)
Palestinian Authority	0.025	(0.026)	Serbia	0.000	(0.018)
Germany	0.054 **	(0.024)	Slovakia	−0.010	(0.024)
Greece	0.026	(0.019)	Vietnam	0.002	(0.015)
Guatemala	−0.002	(0.023)	Slovenia	−0.033	(0.023)
Hong Kong	0.006	(0.026)	Spain	0.024 *	(0.013)
Hungary	0.019	(0.019)	Sweden	−0.019	(0.019)
Iceland	0.028	(0.030)	Switzerland	0.000	(0.020)
Ireland	−0.048 **	(0.020)	Thailand	0.007	(0.017)
Italy	0.021	(0.020)	United Arab Emirates	−0.045 ***	(0.013)
Kosovo	0.022	(0.022)	Turkey	−0.009	(0.018)
Jamaica	0.003	(0.034)	North Macedonia	−0.008	(0.022)
Japan	0.024	(0.021)	United Kingdom	−0.027	(0.024)
Kazakhstan	0.002	(0.011)	Uruguay	−0.006	(0.020)
Jordan	−0.015	(0.026)	Ukrainian regions	0.012	(0.031)

Note. Each row shows the coefficient of the interaction term (Male × Physical Activity), controlling for covariates (Table 3, Column 3), with separate regressions by country. Dependent variable: Life Satisfaction. Two-level HLM (student and school) with robust standard errors (clustered by school) in parentheses. (d) indicates a dummy variable. Regressions weighted by students’ sampling probability. *** *p* < 0.01, ** *p* < 0.05, * *p* < 0.1.

## 5. Discussion

Descriptive analysis shows the average LS score for the sample, with significant gender differences in favour of male participants.

This aligns with existing literature indicating that male adolescents often report higher subjective well-being and LS than their female counterparts [8,13,15]. The positive correlation between economic, social, and cultural status aligns with studies suggesting that higher socioeconomic backgrounds facilitate better well-being outcomes in adolescents due to increased access to resources, amongst others [11,16]. Positive school processes such as climate, teacher support, and the quality of student–teacher relationships, contribute meaningfully to higher LS.

Conversely, bullying victimisation is negatively associated with LS, consistent with extensive research linking adverse peer interactions to lower well-being amongst young people and positive relationships to higher satisfaction well-being [4,23,25]. Intra-class correlation analysis, however, indicates that while both school and country contexts contribute to differences in LS, the country-level context, such as socio-economic conditions and cultural norms, plays a more substantial role in influencing LS in adolescence [11,17]. This underlines the need for initiatives to promote well-being to take a multi-systemic approach targeting the various systems impinging on the well-being of adolescents, such as socio-economic conditions, cultural background, family, peer group, and the school community.

PA reveals a similarly notable consistent gender disparity, with males engaging in more weekly PA than females The overall average of 4.53 h of PA per week reflects a moderate engagement in PA, but is still short of the WHO recommendation of 60 min of moderate to vigorous PA daily. In the case of female adolescents, PA is considerably below average, going down to 2 h per week in some cases, with the lowest being 1.929 amongst Maltese female adolescents. These patterns are consistent with existing literature, which often shows greater engagement in PA among male adolescents [43].

A country analysis of LS and PA by gender shows that, in most of the countries, male adolescents report higher levels of LS and PA compared to their female counterparts. The gender gap in LS is more pronounced in some countries, such as Germany, Ireland and the Netherlands, Qatar and Saudi Arabia, and Chile and the Dominican Republic, reflecting cultural and social norms. On the other hand, in some countries, particularly the Baltic countries, Finland, Sweden, Iceland, and the UK, there is an opposite trend, or the difference is marginal. The gender differences in PA are even more pronounced across most countries, with striking differences in countries such as Malta, Hungary, Ireland, Czech Republic, Peru, Chile and Saudi Arabia. Such country variations in PA might be influenced by factors such as societal attitudes towards female sports participation, the availability of resources, and support for youth PA in general amongst others [41].

The gender differences in both LS and PA indicate that female adolescents may be more at risk in their positive psychological development and well-being. This is in line with other studies which show that adolescent females are less likely to engage in physical exercise and may face more barriers in doing so in some contexts [41,43]. Promoting PA and sports among female adolescents, particularly in contexts where they face barriers to PA engagement and sports participation [43], could be a promising strategy to enhance their positive development and operate as a preventive, resilience-enhancing process against mental health issues. The gender differences also underscore the importance of designing targeted interventions that account for cultural context and gender-specific needs, especially in countries where the gender disparity is substantial [43].

In response to the first research question, multilevel regressions reveal a positive and statistically significant relationship between PA and LS, reinforcing the view that regular PA contributes positively to adolescents’ subjective well-being [29,39]. This finding supports other studies, including longitudinal studies, which show that PA amongst adolescents helps to prevent and reduce mental health issues such as anxiety, depression, loneliness, substance use and anti-social behaviour [27,28,29,37,38]. PA also benefits the physical health of adolescents such as bone health, muscle and motor development, and brain and cognitive development, while preventing obesity which is a risk factor for cardiovascular problems and diabetes amongst others [57]. It is also indicative that PA in adolescence is linked with healthier trajectories in adulthood, such as better mental health and well-being and decreased risk for chronic physical illness [36,58].

The relationship between PA and LS holds for all countries examined, underlining the universal benefits of PA for adolescents’ well-being across cultural contexts and regions. The strength of the association, however, varies across countries, with some nations displaying a more robust relationship between PA and LS than others. For instance, countries like Iceland, Finland and New Zealand demonstrate particularly strong coefficients, indicating that adolescents in these countries experience higher levels of LS as they engage more in PA. On the other hand, the coefficients are relatively weaker in other countries such as Chinese Taipei, Qatar, Saudi Arabia, and Vietnam. These variations may reflect differences in national attitudes toward PA, cultural norms around exercise, or the availability and quality of sports infrastructure and Programmes for adolescents amongst others. Such contextual factors likely play a role in how effectively PA contributes to LS across countries and would warrant further investigation on how to encourage young people to engage in regular physical exercise. Additionally, controlling for covariates like socioeconomic status and school environment strengthens the relationship between LS and PA, underlining that this relationship is not merely a reflection of socioeconomic advantages or supportive school climates but rather an independent contributor to well-being [29,39].

With regard to the second research question, overall, the analysis did not find any moderating role of gender on the PA-LS relationship, indicating that the positive association between PA and LS is consistent across genders, with both male and female adolescents similarly benefitting in LS from PA. This finding aligns with prior research indicating the universal benefits of PA on well-being across genders [13,40]. It underlines the key role of PA in the LS of both male and female adolescents and may explain the lower rates of LS amongst female adolescents. However, in a small number of countries, the analysis identified a significant gender moderation effect, underlining that cultural and contextual factors may influence how gender interacts with PA to affect LS. For instance, societal norms around gender roles and PA may be more or less supportive in certain countries, which could enhance or diminish the psychological benefits of PA for each gender [41,43]. These findings imply that while a general approach to promoting PA is indicated, country-specific interventions might be necessary to account for these cultural nuances.

### 5.1. Implications

The consistent findings from over 60 countries in diverse regions of the world contribute to a broader understanding of well-being in adolescence, highlighting the potential for PA to serve as a universally beneficial intervention to improve LS among adolescents. Parallel to play in childhood, PA may be thus considered as a key developmental goal in adolescence, necessary for well-being and mental health, apart from the more apparent physical health benefits. It may also constitute a positive adolescence experience for mental health and well-being outcomes in adulthood, though this warrants further investigation [59]. The need for PA to be ingrained in the daily life of adolescents has become more critical in view of current trends in their lives, such as increasing urbanization, increased screen time and time on social media, and a more sedentary lifestyle. Moreover, the relatively low level of PA among adolescents in most of the countries in the study, is supported by other cross-national studies. A recent study in more than 40 countries across Europe, North America, and Central Asia [60] reported widespread insufficient levels of moderate to vigorous PA among adolescents, with notable disparities across gender, age, and socio-economic status. On average, nearly a quarter of adolescents were found to be highly physically inactive. Similarly, in their analysis of 298 school-based surveys from 146 countries, including 1.6 million students aged 11–17, Guthold et al. [61] reported that 81% of students globally were insufficiently physically active, with a marked disparity favouring boys over girls.

These findings have clear implications for PA to become more deeply rooted in the daily life of adolescents as a key ingredient for their physical, cognitive, social and emotional development. The various systems in adolescents’ lives such as families, local communities and schools, have a crucial role in encouraging adolescents to engage more frequently and regularly in PA. Schools in particular have access to practically all adolescents for an extended period of time, serving as developmental hubs beyond the family and community. They are thus called to give more priority to physical education as a key educational goal. Increasing time in physical education classes in school improves physical health [30], reduces aggressive behaviour [33], enhances concentration, academic learning and achievement [34,35] and improves social competence and relationships [36]. In a toolkit on PA for schools, the World Health Organisation [62] provides a comprehensive guide for schools on how to foster a culture of PA within a whole school approach, which includes not only quality physical education (minimum 2 h per week), but also active travel to and from school, active before- and after-school programmes, PA during recess and recreation time, active classrooms in school curricula, and inclusive PA for children with individual educational needs.

Whilst underlining the need for a universal approach to promote PA amongst adolescents across the globe, the results underscore that such a global initiative needs to accommodate for both gender and contextual differences to ensure gender-sensitive and contextually adaptive interventions. While the study shows a common cross-cultural pattern in the LS and PA of adolescents in various countries across the world, the cultural variations in some of the findings indicate the need for more investigation into cultural norms and barriers in LS and PA in adolescence.

### 5.2. Limitations and Areas for Further Research

The present study presents several limitations that need to be taken into account when considering the findings and their implications. First, although the study covers a broader geographical range than previous research, the PISA sample is still limited by the underrepresentation or exclusion of emerging and low-income countries, particularly those in Africa [63]. Moreover, not all the countries participating in the PISA study completed the values related to LS and/or PA, which reduced our sample. A more representative sample at global and regional levels would add strength to the study’s findings and provide additional insights into the contextual and cultural determinants of LS and PA in adolescence. Secondly, the study is focused only on one outcome variable, namely LS, or the cognitive aspect of subjective well-being, based on a single-item measure. While this approach is widely used in international large-scale assessments, it inevitably provides a partial perspective on adolescents’ well-being. Future studies could benefit from incorporating a broader range of indicators, including the psychological and affective dimensions of subjective well-being [4]. Thirdly, exploring gender as a moderating variable was restricted to the binary classification (female/male) provided by PISA 2020 [44]; future research should cover a broader spectrum of gender identities. Fourthly, the PISA survey is limited to 15–16-year-old adolescents. While this constitutes a relatively narrow definition of adolescence, it remains the most comprehensive large-scale comparable data currently available to explore the relationship between LS and PA among adolescents. Future studies may investigate this relationship in adolescents from other age groups. Fifthly, this study was based on a cross-sectional design; therefore, no causal but a statistical relationship between LS and PA can be inferred. This means that the direction of the relationship cannot be determined, and it is also plausible that the link operates in both directions. Future studies employing longitudinal or experimental designs would be valuable to clarify the causal pathways underlying this association. Finally, PA in the present study was measured according to the frequency of PA or sports during a typical school week. Unfortunately, PISA does not differentiate between the type, intensity, or context of PA. While it is true that the frequency of PA does not fully capture PA, this measurement based on weekly PA has been used in previous empirical studies (e.g., see, for example, [64,65,66]. Moreover, since cross-national large-scale assessments on the relationship between adolescents’ PA, LS and the moderating role of gender among adolescents are very limited, we believe our study can still offer valuable empirical evidence to the field. Future studies could explore how the relationship between LS and PA may vary according to the type, intensity, or context of PA. For instance, while various forms of PA, including outdoor play, individual exercise, and sports participation, are beneficial for adolescent well-being, evidence suggests that sports participation may offer additional advantages due to its inherently social nature. The well-being and LS benefits of PA are most likely to be realised when the activity is enjoyable and occurs within a supportive social environment [31].

## Figures and Tables

**Table 1:** Descriptive Statistics.

Variables	Mean	SD	Min	Max	Share of Missing Values (%)
Life satisfaction	6.902	2.654	0	10	15.75
Physical activity	4.527	3.626	0	10	6.50
Male (d)	0.504	0.500	0	1	0.01
Age	15.813	0.292	15.170	16.420	0
Foreigner (d)	0.395	0.195	0	1	4.57
Economic Social and Cultural Status	−0.688	1.238	−6.841	7.380	4.15
Being bullied	−0.245	1.060	−1.228	4.694	7.46
Disciplinary climate	0.077	0.993	−2.603	2.226	11.55
Teacher support	0.194	1.118	−3.254	1.649	15.64
Quality of student–teacher relationships	0.095	1.019	−10.090	3.417	19.54
GDP per capita	30,480	20,540	4531	113,871	0

Note. (d) indicates a dummy variable. Statistics calculated using students’ weights. *n* = 613,744.

**Table 2:** Average Life Satisfaction and Physical Activity by Gender. Analysis by country.

Country	Girls		Boys		Country	Girls		Boys	
	LS	PA	LS	PA		LS	PA	LS	PA
Albania	7.741	4.459	8.268	5.796	South Korea	6.543	2.374	-	5.104
Qatar	6.568	4.09	7.131	5.989	Latvia	8.153	4.659	6.952	5.222
Argentina	6.066	4.342	7.328	5.933	Lithuania	7.466	3.707	6.165	5.069
Austria	6.214	3.423	-	4.894	Malta	5.989	1.929	6.861	5.494
Brazil	6.705	3.417	7.154	4.855	Mexico	7.487	4.06	6.977	4.197
Bulgaria	6.418	3.435	0	5.105	Mongolia	6.638	4.384	8.658	6.484
Chile	5.41	2.815	7.342	5.289	Moldova	6.376	3.796	7.869	5.912
Chinese Taipei	6.535	3.404	7.286	5.339	Montenegro	6.156	2.632	6.697	3.909
Colombia	5.8	3.382	6.289	4.992	Netherlands	6.529	3.802	8.247	5.61
Croatia	6.565	4.212	-	5.045	New Zealand	6.664	4.281	7.658	5.98
Czech Republic	6.828	2.21	7.576	5.019	Panama	6.794	4.627	7.149	5.416
Denmark	5.936	3.37	6.992	5.989	Paraguay	7.265	4.276	6.65	4.236
Dominican Republic	5.892	3.677	7.39	6.253	Peru	5.883	2.862	6.982	6.548
El Salvador	6.694	3.671	7.799	3.36	Poland	6.978	4.731	7.322	6.312
Estonia	7.086	4.444	7.159	4.264	Portugal	6.968	4.307	7.783	6.315
Finland	6.435	3.695	7.106	4.931	Qatar	6.292	3.498	8.016	5.867
France	6.392	3.674	7.667	4.838	Romania	6.958	3.36	6.571	4.739
Georgia	6.811	4.468	7.832	6.183	Saudi Arabia	5.778	3.82	8.047	6.559
Palestinian Authority	6.403	3.11	7.325	5.383	Serbia	6.754	4.661	7.424	5.608
Germany	5.609	2.855	7.406	4.911	Slovakia	5.839	4.841	6.982	5.381
Greece	7.255	3.981	7.986	5.4	Vietnam	6.906	3.815	7.611	4.653
Guatemala	6.135	3.998	7.153	4.849	Slovenia	5.661	3.216	-	5.197
Hong Kong	7.456	4.744	6.51	4.666	Spain	6.61	3.635	6.774	4.888
Hungary	6.300	2.127	7.973	6.137	Sweden	7.035	4.358	7.312	6.422
Iceland	6.779	3.697	7.094	5.767	Switzerland	7.071	4.341	6.893	6.515
Ireland	6.684	3.573	8.007	6.084	Thailand	6.656	3.97	7.045	5.326
Italy	6.889	3.383	6.66	3.583	United Arab Emirates	6.408	3.749	6.838	4.781
Kosovo	6.273	3.922	7.924	5.549	Turkey	7.041	4.598	7.493	5.425
Jamaica	6.362	4.303	7.744	4.982	North Macedonia	7.188	3.174	7.632	6.366
Japan	6.125	3.473	7.576	5.328	United Kingdom	7.259	4.174	7.103	5.716
Kazakhstan	5.563	2.883	6.884	5.502	Uruguay	7.01	4.825	6.886	5.479
Jordan	6.684	4.06	7.441	5.82	Ukrainian regions	7.089	4.299	7.161	6.059

Note. Statistics were calculated using students’ weights. LS: Life Satisfaction. PA: Physical Activity.

**Table 3:** Life Satisfaction, Physical Activity and the Moderating Role of Gender. Analysis of the pooled sample.

	(1)	(2)	(3)
Intercept	7.010 ***	7.469 ***	7.486 ***
	(0.070)	(0.428)	(0.432)
Physical activity		0.070 ***	0.066 ***
		(0.003)	(0.004)
Male (d)		0.675 ***	0.640 ***
		(0.058)	(0.053)
Age		−0.062 **	−0.062 **
		(0.027)	(0.027)
Foreigner (d)		−0.045	−0.045
		(0.047)	(0.047)
Economic Social and Cultural Status		0.151 ***	0.151 ***
		(0.017)	(0.017)
Being bullied		−0.489 ***	−0.489 ***
		(0.021)	(0.021)
Disciplinary climate		0.151 ***	0.151 ***
		(0.014)	(0.014)
Teacher support		0.148 ***	0.148 ***
		(0.014)	(0.014)
Quality of student–teacher relationships		0.388 ***	0.388 ***
		(0.021)	(0.021)
GDP per capita		−0.000 ***	−0.000 ***
		(0.000)	(0.000)
Male× Physical activity			0.008
			(0.005)
Random-effects parameters			
	0.552	0.468	0.468
Country: Identity sd(_cons)	(0.065)	(0.061)	(0.061)
	0.767	0.771	0.771
School: Identity sd(_cons)	(0.026)	(0.034)	(0.034)
	2.504	2.224	2.224
sd(Residual)	(0.041)	(0.048)	(0.048)
Intraclass-correlation coefficient			
School	0.045	0.038	0.038
Country	0.146	0.141	0.141
Number of observations			
Students	399,794	399,794	399,794
Schools	16,997	16,997	16,997
Countries	64	64	64

Note. Dependent variable: LS. Three-level HLM (student, school and country) with robust standard errors (clustered by country) in parentheses. (d) indicates a dummy variable. Regressions weighted by students’ sampling probability. *** *p* < 0.01, ** *p* < 0.05 .

**Table 4:** LS and Physical Activity. Analysis by Country.

Country	Coeff.	Std. Err.	Country	Coeff.	Std. Err.
Albania	0.064 ***	(0.012)	South Korea	0.052 ***	(0.014)
Qatar	0.078 ***	(0.014)	Latvia	0.095 ***	(0.010)
Argentina	0.058 ***	(0.011)	Lithuania	0.097 ***	(0.009)
Austria	0.077 ***	(0.013)	Malta	0.118 ***	(0.019)
Brazil	0.080 ***	(0.009)	Mexico	0.078 ***	(0.010)
Bulgaria	0.084 ***	(0.010)	Mongolia	0.086 ***	(0.009)
Chile	0.083 ***	(0.011)	Moldova	0.056 ***	(0.012)
Chinese Taipei	0.049 ***	(0.009)	Montenegro	0.067 ***	(0.014)
Colombia	0.054 ***	(0.011)	Netherlands	0.054 ***	(0.010)
Croatia	0.084 ***	(0.009)	New Zealand	0.109 ***	(0.011)
Czech Republic	0.089 ***	(0.011)	Panama	0.054 ***	(0.015)
Denmark	0.070 ***	(0.012)	Paraguay	0.070 ***	(0.011)
Dominican Republic	0.061 ***	(0.011)	Peru	0.092 ***	(0.011)
El Salvador	0.068 ***	(0.011)	Poland	0.108 ***	(0.013)
Estonia	0.084 ***	(0.010)	Portugal	0.073 ***	(0.009)
Finland	0.109 ***	(0.009)	Qatar	0.045 ***	(0.012)
France	0.067 ***	(0.010)	Romania	0.053 ***	(0.008)
Georgia	0.057 ***	(0.011)	Saudi Arabia	0.053 ***	(0.011)
Palestinian Authority	0.058 ***	(0.012)	Serbia	0.087 ***	(0.009)
Germany	0.076 ***	(0.012)	Slovakia	0.085 ***	(0.012)
Greece	0.084 ***	(0.011)	Vietnam	0.052 ***	(0.009)
Guatemala	0.075 ***	(0.012)	Slovenia	0.084 ***	(0.013)
Hong Kong	0.066 ***	(0.011)	Spain	0.080 ***	(0.007)
Hungary	0.062 ***	(0.010)	Sweden	0.083 ***	(0.010)
Iceland	0.134 ***	(0.014)	Switzerland	0.059 ***	(0.012)
Ireland	0.089 ***	(0.010)	Thailand	0.069 ***	(0.009)
Italy	0.071 ***	(0.010)	United Arab Emirates	0.091 ***	(0.007)
Kosovo	0.079 ***	(0.010)	Turkey	0.074 ***	(0.010)
Jamaica	0.079 ***	(0.020)	North Macedonia	0.090 ***	(0.010)
Japan	0.056 ***	(0.009)	United Kingdom	0.081 ***	(0.013)
Kazakhstan	0.056 ***	(0.005)	Uruguay	0.083 ***	(0.010)
Jordan	0.075 ***	(0.012)	Ukrainian regions	0.088 ***	(0.014)

Note. Each row shows the coefficient of physical activity, controlling for covariates (Table 3, Column 2), with separate regressions by country. Dependent variable: Life Satisfaction. Two-level HLM (student and school) with robust standard errors (clustered by school) in parentheses. (d) indicates a dummy variable. Regressions weighted by students’ sampling probability. *** *p* < 0.01 .

## Data Availability

The data used in this study are publicly available and can be accessed at the following link: https://www.oecd.org/en/data/datasets/pisa-2022-database.html accessed on 30 May 2025.

## Abbreviations

The following abbreviations are used in this manuscript:

LS	Life Satisfaction
PA	Physical Activity
PISA	Programme for International Student Assessment
OECD	Organization for Economic Co-operation and Development
WHO	World Health Organization

## Appendix A

**Table A1:** List of Participating Countries and Economies.

Albania	South Korea
Qatar	Latvia
Argentina	Lithuania
Austria	Malta
Brazil	Mexico
Bulgaria	Mongolia
Chile	Moldova
Chinese Taipei	Montenegro
Colombia	Netherlands
Croatia	New Zealand
Czech Republic	Panama
Denmark	Paraguay
Dominican Republic	Peru
El Salvador	Poland
Estonia	Portugal
Finland	Qatar
France	Romania
Georgia	Saudi Arabia
Palestinian Authority	Serbia
Germany	Slovakia
Greece	Vietnam
Guatemala	Slovenia
Hong Kong	Spain
Hungary	Sweden
Iceland	Switzerland
Ireland	Thailand
Italy	United Arab Emirates
Kosovo	Turkey
Jamaica	North Macedonia
Japan	United Kingdom
Kazakhstan	Uruguay
Jordan	Ukrainian regions

**Table A2:** Definition of the control variables.

**Variable**	**Definition**
Age	Age was defined by the age of the student in years. PISA calculates this variable as the difference between the year and month of the testing and the year and month of a student’s birth, which was obtained from school records from the student sampling data and validated by comparing to the students’ responses in the questionnaire.
Being bullied	Being bullied index was derived from students’ responses of how often (“Never or almost never”, “A few times a year”, “A few times a month”, “Once a week or more”) they had a range of experiences at school that are indicative of being bullied during the past 12 months: “Other students made fun of me”; “I was threatened by other students”; “Other students took away or destroyed things that belonged to me”; “I got hit or pushed around by other students”; “Other students spread nasty rumours about me”; “I was in a physical fight on school property”; “I stayed home from school because I felt unsafe”; “I gave money to someone at school because they threatened me”.
Disciplinary climate	Disciplinary climate index was derived from students’ responses about how often (“every lesson”, “most lessons”, “some lessons”, “never or hardly ever”) the following things happened: “Students do not listen to what the teacher said.”; “There is noise and disorder”; “The teacher has to wait a long time for students to quiet down”; “Students cannot work well.”; “Students do not start working for a long time after the lesson begins”;” Students get distracted by using [digital resources] (e.g., smartphones, websites, apps)”; “Students get distracted by other students who are using [digital resources] (e.g., smartphones, websites)”.
Economic, social, and cultural status	Economic, social, and cultural status index was derived from three indicators: parents’ highest level of education in years (PAREDINT), parents’ highest occupational status (HISEI), and home possessions (HOMEPOS). PISA 2022 Technical Report (pp. 407–409) provides a detailed description of this index and its three indicators.
Foreigner	Foreigner was defined using the PISA dummy variable “In what country were you born?” equal to one if the student’s response was “Other country” and equal to zero if the student’s response was “Country of test.”
Male	Male was defined using the PISA dummy variable “Are you female or male?”.
Quality of student–teacher relationships	Quality of student–teacher relationships index was derived from students’ ratings of their agreement (“Strongly disagree”, “Disagree”, “Agree”, “Strongly agree”) with the eight following statements: “The teachers at my school are respectful towards me”; “If I walked into my classes upset, my teachers would be concerned about me”; “If I came back to visit my school 3 years from now, my teachers would be excited to see me”; “I feel intimidated by the teachers at my school”; “When my teachers ask how I am doing, they are really interested in my answer”; “The teachers at my school are friendly towards me”; “The teachers at my school are interested in students’ well-being”; “The teachers at my school are mean towards me”.
Teacher support	Teacher support index was derived from students’ responses about how often (“every lesson”, “most lessons”, “some lessons”, “never or almost ever”) the following things happened in their language-of-instruction maths lessons: “The teacher shows an interest in every student’s learning.”; “The teacher gives extra help when students need it.”; “The teacher helps students with their learning.”; “The teacher continues teaching until the students understand.

Note. All PISA variables were constructed and provided by the PISA team. This table provides detailed information about their definition. More details about the design, construction, and psychometric properties can be found in the PISA 2022 Technical Report [44].

**Table A3:** Cronbach’s alpha for the IRT-based variables included in the study.

Country/Economy (Language Group)	Being Bullied	Disciplinary Climate	Teacher Support	Quality of Student–Teacher Relationships
Albania (Albanian)	0.87	0.89	0.92	0.71
Argentina (Spanish)	0.75	0.87	0.91	0.70
Austria (German)	0.80	0.85	0.89	0.75
Brazil (Portuguese)	0.78	0.85	0.90	0.67
Bulgaria (Bulgarian)	0.85	0.89	0.91	0.72
Chile (Spanish)	0.76	0.87	0.93	0.76
Chinese Taipei (Chinese)	0.77	0.87	0.91	0.79
Colombia (Spanish)	0.75	0.86	0.92	0.74
Croatia (Croatian)	0.80	0.88	0.90	0.72
Czech Republic (Czech)	0.78	0.86	0.89	0.70
Denmark (Danish)	0.78	0.83	0.89	0.75
Dominican Republic (Spanish)	0.76	0.86	0.91	0.76
El Salvador (Spanish)	0.79	0.87	0.93	0.78
Estonia (Estonian)	0.77	0.88	0.89	0.73
Estonia (Russian)	0.78	0.86	0.84	0.76
Finland (Finnish, Swedish)	0.83	0.88	0.90	0.76
France (French)	0.78	0.85	0.89	0.65
Georgia (Georgian, Azerbaijani, Russian)	0.84	0.88	0.88	0.67
Germany (German)	0.77	0.85	0.88	0.74
Greece (Greek)	0.79	0.81	0.87	0.72
Guatemala (Spanish)	0.77	0.82	0.89	0.70
Hong Kong—China (Chinese)	0.84	0.88	0.93	0.76
Hong Kong—China (English)	0.83	0.89	0.90	0.69
Hungary (Hungarian)	0.80	0.89	0.89	0.76
Iceland (Icelandic)	0.83	0.89	0.91	0.75
Ireland (English, Irish)	0.78	0.86	0.91	0.73
Italy (Italian, German)	0.76	0.86	0.90	0.71
Jamaica (English)	0.74	0.84	0.90	0.67
Japan (Japanese)	0.73	0.78	0.92	0.76
Jordan (Arabic)	0.87	0.87	0.89	0.66
Kazakhstan (Kazakh)	0.71	0.87	0.90	0.71
Kazakhstan (Russian)	0.74	0.89	0.91	0.77
Latvia (Latvian)	0.76	0.85	0.87	0.74
Latvia (Russian)	0.80	0.87	0.86	0.76
Lithuania (Lithuanian, Russian, Polish)	0.84	0.90	0.90	0.76
Malta (English)	0.82	0.84	0.91	0.74
Mexico (Spanish)	0.76	0.86	0.92	0.72
Mongolia (Mongolian, Kazakh)	0.80	0.85	0.91	0.67
Montenegro (Montenegrin, Albanian)	0.80	0.88	0.92	0.72
Netherlands (Dutch)	0.83	0.86	0.87	0.69
New Zealand (English)	0.81	0.88	0.92	0.72
North Macedonia (Albanian)	0.86	0.89	0.92	0.66
North Macedonia (Macedonian)	0.84	0.89	0.92	0.71
Panama (Spanish, English)	0.74	0.84	0.91	0.69
Paraguay (Spanish)	0.77	0.86	0.89	0.68
Peru (Spanish)	0.74	0.84	0.91	0.73
Poland (Polish)	0.83	0.89	0.89	0.73
Portugal (Portuguese)	0.80	0.86	0.93	0.72
Qatar (Arabic)	0.89	0.89	0.93	0.66
Qatar (English)	0.82	0.84	0.91	0.70
Republic of Moldova (Romanian)	0.80	0.88	0.90	0.74
Republic of Moldova (Russian)	0.80	0.90	0.87	0.76
Romania (Romanian, Hungarian)	0.81	0.88	0.89	0.72
Saudi Arabia (Arabic, English)	0.85	0.89	0.92	0.69
Serbia (Serbian, Hungarian)	0.83	0.88	0.92	0.73
Slovak Republic (Slovak, Hungarian)	0.83	0.86	0.90	0.73
Slovenia (Slovenian)	0.86	0.88	0.89	0.74
Slovenia (Slovenian-ISCED2)	0.82	0.83	0.87	0.70
Spain (Catalan)	0.80	0.85	0.91	0.74
Spain (Spanish, Galician, Basque, Valencian)	0.77	0.84	0.91	0.74
Sweden (Swedish, English)	0.83	0.87	0.90	0.76
Switzerland (French)	0.80	0.83	0.89	0.69
Switzerland (German, Italian)	0.81	0.83	0.88	0.76
Thailand (Thai)	0.86	0.89	0.93	0.68
Türkiye (Turkish)	0.78	0.84	0.90	0.76
United Arab Emirates (Arabic)	0.90	0.90	0.93	0.68
United Arab Emirates (English)	0.88	0.87	0.91	0.67
United Kingdom (English, Welsh)	0.81	0.89	0.92	0.73
Uruguay (Spanish)	0.77	0.86	0.92	0.70
Viet Nam (Vietnamese)	0.76	0.83	0.89	0.73

Source. PISA 2022 Technical Report (See Appendix A Table A1, available at: https://stat.link/v6uq1n accessed on 30 May 2025) [44].

**Table A4:** Matrix correlation.

		(1)	(2)	(3)	(4)	(5)	(6)	(7)	(8)	(9)	(10)	(11)
(1)	Life Satisfaction	1										
(2)	Physical activity	0.165 ***	1									
(3)	Male (d)	0.143 ***	0.232 ***	1								
(4)	Age	−0.007	−0.002	0.002	1							
(5)	Foreigner (d)	−0.030 ***	−0.001	0.008	0.012 *	1						
(6)	Economic Social and Cultural Status	0.028 ***	0.040 ***	0.020 ***	−0.014 *	0.063 ***	1					
(7)	Being bullied	−0.210 ***	0.021 ***	0.055 ***	−0.019 *	0.034 **	−0.021 *	1				
(8)	Disciplinary climate	0.121 ***	−0.002	−0.053 ***	0.014 *	−0.003	0.037 ***	−0.250 ***	1			
(9)	Teacher support	0.158 ***	0.026 ***	−0.012	−0.003	0.011 *	−0.083 ***	−0.129 ***	0.143 ***	1		
(10)	Quality of student–teacher relationships	0.221 ***	0.039 ***	−0.021 ***	0.009 *	0.001	−0.019 *	−0.189 ***	0.200 ***	0.351 ***	1	
(11)	GDP per capita	−0.068 ***	−0.065 ***	0.012 *	−0.006	0.232 ***	0.377 ***	0.002	0.029 ***	−0.064 ***	−0.035 ***	1

(d) indicates a dummy variable; *** *p* < 0.01, ** *p* < 0.05, * *p* < 0.1.

**Table A5:** Variance Inflation Factor (VIF) and Tolerance Values for Predictor Variables in the Analysis.

	VIF	1/VIF
Physical activity	1.070	0.936
Male (d)	1.060	0.941
Age	1.000	0.999
Foreigner (d)	1.070	0.932
Economic Social and Cultural Status	1.120	0.889
Being bullied	1.100	0.911
Disciplinary climate	1.110	0.902
Teacher support	1.170	0.858
Quality of student–teacher relationships	1.190	0.841
GDP per capita	1.200	0.835
Mean VIF	1.110	

(d) indicates a dummy variable.
